# The establishment of the objective diagnostic markers and personalized medical intervention in patients with major depressive disorder: rationale and protocol

**DOI:** 10.1186/s12888-016-0953-z

**Published:** 2016-07-15

**Authors:** Xiaozhen Lv, Tianmei Si, Gang Wang, Huali Wang, Qi Liu, Changqing Hu, Jing Wang, Yunai Su, Yu Huang, Hui Jiang, Xin Yu

**Affiliations:** Peking University Sixth Hospital (Institute of Mental Health), Beijing, China; National Clinical Research Center for Mental Disorders & Key Laboratory for Mental Health, Ministry of Health, Peking University, Beijing, China; Beijing Anding Hospital, Capital Medical University, Beijing, China; National Engineering Research Center for Software Engineering, Peking University, Beijing, China

**Keywords:** Major depressive disorder, Subtype, Diagnostic, Personalized treatment

## Abstract

**Background:**

Major depressive disorders (MDD) is a common mental disorder with high prevalence, frequent relapse and associated with heavy disease burden. Heritability, environment and their interaction play important roles in the development of MDD. MDD patients usually display a wide variation in clinical symptoms and signs, while the diagnosis of MDD is relatively subjective. The treatment response varies substantially between different subtypes of MDD patients and only half respond adequately to the first antidepressant. This study aims to define subtypes of MDD, develop multi-dimension diagnostic test and combined predictors for improving the diagnostic accuracy and promoting personalized intervention in MDD patients.

**Methods/Design:**

This is a multi-center, multi-stage and prospective study. The first stage of this study is a case–control study, aims to explore the risk factors for developing MDD and then define the subtypes of MDD using 1200 MDD patients and 1200 healthy controls with a set of questionnaire. The second stage is a diagnostic test, aims to indentify and replicate the potential indicators to assist MDD diagnosis using 600 MDD patients and 300 healthy controls from the first stage with a set of questionnaire, neuropsychological assessment and a series of biomarkers. The third stage is a 96-week longitudinal study, including 8-week acute period treatment and 88-week stable period treatment, aims to identify overall predictors of treatment effectiveness on MDD at week 8 post treatment and to explore the predictors on MDD prognosis in the following 2 years using 600 MDD patients from the first stage with a set of questionnaire, neuropsychological assessment and a series of biomarkers. The primary outcome measure is the change of the total score of 17-Item Hamilton Rating Scale for Depression.

**Discussion:**

This study will provide strong and suitable evidence for enhancing the accuracy of MDD diagnosis and promoting personalized treatment for MDD patients in clinical practice.

**Trial registration:**

ClinicalTrials.gov: NCT02023567; registration date: December 2013.

## Background

Major depressive disorders (MDD) is a common, complex, often difficult-to-treat and high-relapse clinical condition [1]. MDD causes the largest amount of disability, accounting for almost 12 % of all total years lived with disability worldwide [2]. MDD was also a contributor of burden allocated to suicide and ischemic heart disease [3]. The pain and suffering of individuals with MDD and those close to them result in a heavy economic toll to this country in terms of both treatment costs and lost productivity [4]. China is also confronted with this daunting challenges against MDD. It was assessed that the 1-month prevalence of mood disorders (mainly MDD) was 6.1 % [5].

MDD commonly arises when a vulnerable individual confronts adversity. MDD is familial, with heritability estimated to be 0.37. Environmental influences specific to an individual are also etiologically significant [6, 7]. Genetic factors partially influence overall risk of illness, but also influence the sensitivity of individuals to the depression-inducing effects of environmental adversity. The interaction of genotype and environment is significant in the prediction of onset of MDD [8]**.** Despite decades of research, there remains little consensus on how to distinguish between MDD subtypes. Loo et al. examined the evidence for the existence of data-driven symptomatic subtypes of depression and did not provide conclusive evidence for the existence of depressive symptom dimensions or symptomatic subtypes [9]. A persistent theme in the debate on the classification of MDD has been the question of how to distinguish biological depressions from depressions that are social in origin. It appears to be “ at least partly” to distinguish those individuals whose depressive illness is largely “genetic” versus “environmental” [10]. One the other side, some distinct subtypes had been suggested and found subtypes associated with treatment outcomes [11–13]. For optimized treatment, it is probably meaningful to classify MDD into different subtypes basing on its psychopathology.

The diagnosis of MDD is based on relatively subjective interviews and questionnaires for assessments of symptoms. However, affected individuals display quite a wide variation in clinical symptoms and signs [10]. Even in some high-income countries, people who are depressed are not always correctly diagnosed [1]. Biomarkers in psychiatry present a promising addition to advance the diagnosis, treatment and prevention of psychiatric diseases [14]. The development of reliable diagnostic tests using biomarkers could aid in the diagnosis of MDD. Many peripheral biomarkers, including inflammatory cytokines, immunological markers, growth factors, endocrine factors, metabolic markers and oxidative stress markers, have been investigated because these markers are thought to be involved in the pathophysiology of MDD [14–18]. However, it is clear that even though a large number of biomarkers have been linked to MDD, each individually explains a very modest proportion of the variance in MDD risk. For clinical diagnosis of MDD, all individual marker-based approaches yielded insufficient sensitivity and specificity [19]. It had been reported that a composite, multi-assay diagnostic test for MDD demonstrated adequate sensitivity and specificity (about 91 and 81 %) for American [19]. The results in a Japanese population were not in conformity with those in American [20]. It is needed to confirm the performance of the test in large Chinese sample.

Several treatments for MDD are available, while response to treatments varies substantially between patients and more than half will fail to respond adequately to the first antidepressant they are prescribed [21]. The heterogeneity of treatment effects complicates clinical decision-making. One approach to enhancing treatment outcomes in MDD has been the use of standardized sequential treatment algorithms and measurement-based care. Pretreatment tests that predict which patients will respond to which types of treatment could save time, money and patient burden. A large body of literature reported that some demographic characteristics, clinical symptoms, stress events, genotypes and biomarkers could foretell overall treatment outcome [11–13, 15, 22–37]. However, robust and combined predictors of treatment response remain elusive [7, 38]. It is urgent and critical to combine possible factors to form the basis of new paradigms for antidepressant treatment selection.

## Objectives

This study is designed to verify the role of heritability and environment in the onset of MDD, define the subtypes of MDD and evaluate a range of factors/predictors to aid MDD diagnosis and personalized treatment within MDD patients and healthy controls. The aims of this study are to:

1. Verify the role of heritability and environment in the onset of MDD and define the subtypes of MDD according to the influence of heritability and environment on its development.
2. Identify potential indicators to assist MDD diagnosis basing on the baseline information, neuropsychological assessment and biological markers.
3. Identify overall predictors for treatment effectiveness of selective serotonin reuptake inhibitors (SSRIs) on MDD after up to 8 weeks
4. Explore the predictors on MDD prognosis in the following two years.

## Methods/Design

### Organizational structure and quality control

The infrastructure of this study includes an executive management team, 9 clinical sites at 9 top tertiary hospitals (7 within academic settings and 2 in clinical practices) located in 6 provinces/municipalities and one clinical research organization. Each clinical site has a principal investigator (responsible for all work in this site), one research coordinator (responsible for the coordination in this site/with the executive management team), 2 to 4 investigators (responsible for recruitment, clinical evaluation, neuropsychological assessment, blood specimen collection), one data typist (responsible for entering paper-based questionnaire data into a web-based data system) and one clinical research assistant (CRA) assigned by the clinical research organization (responsible for supervising compliance to study protocol and checking the conformance of the paper-based questionnaire and the web-based).

Each clinical sites are selected based on the likelihood of executing the protocol and their previous research experience. All principal investigators have chaired or participated in some clinical studies. Prior to enrolling participants in each site, work manuals will be supplied to each site to provide instructions on all relevant research issues, including enrollment, clinical evaluation, neuropsychological assessment, serious adverse events reporting procedure, data entering and checking and blood specimen collection, storage and transportation. The researchers are all strictly trained according to the work manual. As a new staff joins in, they will be trained by their principal investigator and research coordinator. Clinical data are acquired by psychiatrists. The investigators will remain in contact with those participants who should be followed up to minimize premature discontinuation. The CRA will visit the research site once a week and feedback the progress and main problems to the principal investigator and the executive management team. A monthly review of the program progress, key problems and advice will be reported to all researchers by the executive management team. In addition, a group of experts assigned by the executive management team will go to each site twice a year to guide and confirm the protocol implementation and assess the reliability of clinical evaluation. Inter-rater reliability for the primary outcome measure (the 17-Item Hamilton Rating Scale for Depression, HRSD_17_) [39] is audited for each psychiatrist who involved in clinical evaluation at each site annually. The executive management team calls for all principal investigators and research coordinators once a year face-to-face to discuss the difficulties and share experience to ensure the protocol implementation and study quality.

### Study design

This is a multi-center, multi-stage and prospective study. It includes 3 stages. (1) The first stage is a case–control study, basing on the baseline information of 1200 MDD patients (1200-MDD patients group) and 1200 health controls (1200-Healthy controls group), aims to explore the risk factors of developing MDD and then define the subtypes of MDD. (2) The second stage is a diagnostic test, basing on the baseline information, neuropsychological assessment and biological markers of 600 MDD patients (600-MDD patients subgroup) and 300 health controls (300-Healthy controls subgroup) from the first stage, aims to indentify and replicate the potential indicators to assist MDD diagnosis. (3) The third stage is a 96-week longitudinal study, including 8-week acute period treatment and 88-week stable period treatment of the 600-MDD patients subgroup. A total of 600 MDD patients from the first stage, who would receive one of 6 SSRIs within the range of does for 8 weeks (fluoxertine hydrochloride 20-60 mg/day, paroxetine hydrochloride 20-60 mg/day, sertraline hydrochloride 50-200 mg/day, citalopram 20-60 mg/day, escitalopram 10-20 mg/day, fluvoxamine 50-300 mg/day) decided by their attending physicians, would enter into the second and third stage. The 600-MDD patients subgroup would receive clinical evaluation at baseline, week 2, 4 and 8 and receive neuropsychological assessment and blood collection at baseline and week 8 meanwhile. The aim of this phase is to identify overall predictors of treatment effectiveness of SSRIs, basing on the baseline information, clinical evaluation, neuropsychological assessment and biological markers of 600-MDD patients subgroup. The 600-MDD patients subgroup would enter into the stable period treatment and be followed up in the following 88 weeks after completing the above acute period treatment. The treatment in the stable period would be completely decided by their attending physicians according to clinical practice. These patients would receive clinical evaluation at week 24, 48 and 96 and receive neuropsychological assessment and blood collection at week 48 meanwhile. The aim of this phase is to explore the predictors for MDD prognosis.

### Participants

The participants are planned to enroll between December 2013 to December 2016. The goal is to recruit 1200 MDD patients and 1200 healthy controls to complete baseline evaluation and 300 out of 1200 healthy controls will meanwhile receive neuropsychological assessment and blood collection. The 600-MDD patients subgroup (600 out of 1200 MDD patients), who meet more strict criteria and will receive one of 6 SSRIs for 8 weeks in acute period as aforementioned, will be followed up for 96 weeks. The details of inclusion and exclusion criteria for each group are listed in Table 1.

**Table 1:** The inclusion and exclusion criteria for MDD patients and healthy controls, respectively

Study group	Inclusion criteria	Exclusion criteria
1200-MDD patients	(1) age between 18 and 55 years at the time of enrollment; (2) diagnosis of MDD based on the Chinese Version of MINI according to DSM-IV TR; (3) first-episode or relapsed; (4) having the ability of reading and writing to complete the questionnaire and psychological assessment; (5) providing written confirmation of informed consent.	(1) lifetime or current diagnosis of other psychotic disorder, alcohol/substances dependence or cognitive impairment; (2) severe somatic diseases, such as severe cardio-cerebral vascular diseases, respiratory diseases, liver diseases, kidney diseases, or malignant tumors; (3) not signed the informed consent; (4) been engaging in other studies.
600-MDD patients subgroup	Besides those same as 1200-MDD patients’, more inclusion criteria are as the follows: (1) first-episode or relapsed more than1 time in the past 3 years and in acute episode period; (2) total score of HRSD_17_ ≥ 14 when being screened; (3) not taking anti-depressant regularly in the past 2 weeks or should change anti-depressant according to the psychiatrist’s advice.	Besides those same as 1200-MDD patients’, more exclusion criteria are as the follows: (1) resistant depression (not improved after taking 2 kinds of anti-depressant with adequate dosage and duration; (2) having history of epilepsy; (3) taking MECT therapy in the past 3 months; (4) pregnant or breast-feeding.
1200-Healthy controls	(1) age between 18 and 55 years at the time of enrollment; (2) providing written confirmation of informed consent prior to engaging the study.	(1) lifetime or current diagnosis of any mental diseases; (2) severe somatic diseases, such as severe cardio-cerebral vascular diseases, respiratory diseases, liver diseases, kidney diseases, or malignant tumors; (3) not signed the informed consent; (4) been engaging other studies.

*Abbreviations*: *MDD* major depressive disorder, *MINI* mini-international neuropsychiatric interview, *DSM-IV TR* diagnostic and statistical manual of mental disorders, fourth edition text revision, *HRSD*_17_ the 17-item Hamilton rating scale for depression, *MECT* modified electric convulsive therapy

### Enrollment, clinical treatment and follow-up

1. For 1200 MDD patients, the potential participants of the outpatients and inpatients in each clinical sites would be screened and enrolled by the investigators according to the inclusion and exclusion criteria of 1200-MDD patients firstly. To ensure representative and reflect the routine clinical practice, the treatment of eligible MDD patients would be decided and adjusted by their attending physicians. The 600-MDD patients subgroup would receive the same type and dose of one of 6 SSRIs as aforementioned in the 8-week acute treatment period. In this period, except additional treatments for comorbid physical diseases or short-lasting benzodiazepines for severe insomnia, other antipsychotic medications, other anti-depressant, mood stabilizer, systematic psychotherapy and long-lasting benzodiazepines were not allowed. The 600-MDD patients subgroup will receive clinical visits at 2nd, 4th, 8th, 24th, 48th and 96th week in the following 96 weeks after baseline evaluation. The primary treatment outcome evaluation index is the change of the total score of HRSD_17_: treatment effective defined as a ≥50 % decrease from the baseline total score of HRSD_17_, clinical recovery defined as a ≥50 % decrease from the baseline total score of HRSD_17_ and the total score of HRSD_17_ < 7, no response defined as a <50 % decrease from the baseline total score of HRSD_17_. The secondary index includes the change of the total score of Hamilton Anxiety Scale (HAMA), Global Impression of Severity (CGI-S), number of MDD patients with serious adverse events. Other 600 MDD patients will complete this study after baseline evaluation.

2. For 1200 healthy controls, except for the researchers involved in this study and the family members of enrolled MDD patients, those working in the clinical sites, the friends of the patients, college students, or the residents near the clinical sites are all potential participants. Those who are interested in this study will contact the investigators and be enrolled according to the inclusion and exclusion criteria of healthy controls. All healthy controls will receive baseline evaluation and 300 out of them will voluntarily receive neuropsychological assessment and supply blood specimen.

**Fig. 1:**
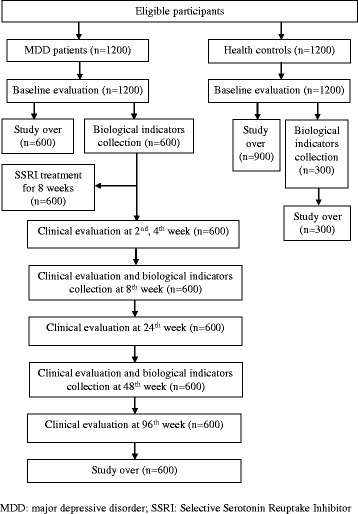
Research flowchart

See “Fig. 1 the research flowchart” for a detailed overview of the research procedure.

## Data collection

### Screening, demographic, clinical and cognitive function data

At screening, the trained investigators gather participant eligibility. The Chinese Version of MINI [40] was used to confirm DSM-IV criteria for MDD [41], and assess for psychiatric and substance abuse disorders and other potential exclusion criteria. After enrollment, data of demographic, medical history, family history of psychiatric disease, MDD course, treatment process, concurrent treatment, stress events, mood disorder episodes and adverse events at baseline and/or follow-up point would be collected using a set of self-designed questionnaire; other data, including life events in the past year, adverse childhood experience, social support, coping style, personality trait, anxiety symptom, depressive symptom severity and improvement would be assessed and acquired using standardized Chinese version of scales, respectively, including Life Events Scale, Childhood Trauma Questionnaire, Social Support Questionnaire, Simplified Coping Style Questionnaire and, Eysenck Personality Questionnaire, Hamilton Anxiety Scale, HRSD_17_ and Clinical Global impression Scale [39, 42].

A series of neuropsychological assessment [43] to reflect cognitive function will be completed at baseline for 600-MDD patients subgroup and 300-Healthy controls subgroup, respectively, and also at 8th and 48th week for 600-MDD patients subgroup, respectively. There are 5 domains and each is assessed by at least one test: attention/vigilance assessed by Continuous Performance Test-Identical Pairs, speed of information processing assessed by Animal Verbal Fluency Scale, Color Trial Test I and II and The Brief Assessment of Cognition in Schizophrenia, visual learning assessed by Brief Visual Memory Test-Revised, verbal learning assessed by Hopkins Verbal Learning Test-Revised and executive function assessed by Stroop Color Word Test. The trained investigator would guide the participants completing the tests according to standardized instructions. Except for the test of Continuous Performance Test-Identical Pairs completed using a standardized computerized touch screen platform, other tests would be completed using standardized scales guiding by the investigator.

The measure, participant, method, assessment time and administrator of the data collection are listed in Table 2.

**Table 2:** Data Collection at Screening, Baseline and Follow-up Evaluation

Domain	Measure	Participant	Method	Assessment time	Administrator
Informed consent	Informed consent	All participants	Interview	Screening	Investigator
Eligibility	Inclusion/Exclusion	All participants	Interview	Screening	Investigator
Psychiatric diagnoses	MINI	All participants	Interview	Screening	Investigator
Demographic, medical history, family history of psychiatric disease and MDD course	Self-designed questionnaire	All participants^a^	Self-report	Baseline	Investigator
Life events in the past year, adverse childhood experience, social support, coping style, personality trait	LES, CTQ, SSQ, SCSQ,EPQ	All participants	Interview	Baseline	Investigator
Treatment process, concurrent treatment, stress events, mood disorder episodes and adverse events	Self-designed questionnaire	600-MDD patients subgroup	Self-report	Every follow-up	Investigator
Anxiety symptom	HAMA	600-MDD patients subgroup	Interview	Every follow-up	Investigator
Depressive symptom severity	HRSD_17_	600-MDD patients subgroup	Interview	Baseline and every follow-up	Investigator
Depressive symptom improvement	CGIS	600-MDD patients subgroup	Interview	Every follow-up	Investigator
Cognitive function	CPT-IP, AVFSS, CTTI and II, BACS, BVNT-R, HVLT-R, SCWT	600-MDD patients and 300-Health controls subgroup	Interview	Baseline, week 8 and 48^b^	Investigator and computerized touch screen platform^c^

*Abbreviations*: *MINI* Mini-International Neuropsychiatric Interview, *LES* Life Events Scale, *CTQ* Childhood Trauma Questionnaire, *SSQ* Social Support Questionnaire, *SCSQ* Simplified Coping Style Questionnaire, *EPQ* Eysenck Personality Questionnaire, *HAMA* Hamilton Anxiety Scale, *HRSD*_17_ 17-Item Hamilton Rating Scale for Depression, CGIS Clinical Global impression Scale, *CPT-IP* Continuous Performance Test-Identical Pairs, *AVFSS* Animal Verbal Fluency Scale, *CTTIand II* = Color Trial Test I and II, *BACS* the Brief Assessment of Cognition in Schizophrenia, *BVNT-R* Brief Visual Memory Test-Revised, *HVLT-R* Hopkins Verbal Learning Test-Revised, *SCWT* Stroop Color Word Test

^a^ Except “MDD course” for 1200 MDD patients only, other data of this domain would be collected for all participants

^b^ For 300-Healthy controls subgroup, cognitive function data would only be collected at baseline

^c^ Except for the data of CPT-IP collected using a standardized computerized touch screen platform, other data of this domain would be collected by the trained investigator

### Molecular data

Blood samples were collected by venepuncture at baseline for 600-MDD patients subgroup and 300-Healthy controls subgroup, respectively, and also at 8th and 48th week for 600-MDD patients subgroup, respectively. Plasma samples were collected in EDTA containing tubes; within 1 h from blood sampling, they will be centrifuged at 3000 rpm for 15 min. Afterward plasma samples were carefully transferred to new tubes and stored at −80°Cuntil further analysis. Serum samples were collected in anticoagulant-free tubes. After centrifugation, serum will be drawn off and frozen at −80°Cuntil needed. Avoid repeated freeze-thaw cycles. Assays will be performed at the central laboratory (Peking University Sixth Hospital). Immune/inflammation and neurotrophic factor pathways have been implicated in the pathogenesis of MDD. The activity of peripheral cytokines correlates with inflammatory processes in the central nervous system [32]. Immune and neurothrophic biomarkers that maybe associated with MDD will be measured in serum or plasma with commercially available kits following the manufacturer’s instructions.

Genomic DNA will be extracted from venous blood using a commercially available QIAamp® DNA Blood Mini Kit. Based on the HapMap database [44] and the NCBI SNP database [45], we selected functional polymorphisms related to depression canidate genes with minor allele frequencies (MAFs) >5 % according to the HapMap database for CHB [44]. Functional polymorphisms will be determined using the F-SNP database [46] which identifies polymorphisms with effects on protein coding, splicing regulation, transcription regulation, and/or post translation. Genotyping will be conducted using the Sequenom MassArray system (Sequenom iPLEX assay) by following the manufacturer’s instructions. Approximately 15 ng of genomic DNA will be used to genotype each sample. Locus-specific PCR and detection primers will be designed using the MassArray Assay Design 3.0 software (Sequenom). The DNA samples will be amplified by multiplex PCR reactions, and the PCR products will then be used for locus-specific single-base extension reaction. The resulting products will be desalted and transferred to a 384-element SpectroCHIP array. Allele detection will be performed using MALDI-TOF MS spectroscopy. The mass spectrograms will be analyzed by the MassArray TYPER software (Sequenom). To assess genotyping quality, 3.5 % of samples will be genotyped twice.

### Sample size, power and effect size

The first goal of this study is to verify the role of heritability and environment in the onset of MDD and identify a number of characteristics for defining the subtypes of MDD. The study design for this part is a case–control study. The sample size has been selected to detect small effects for factors at odds ratio 1.5, if the proportion of factors in control group more than10%, with a power of 0.85 and a two-tailed significance level at 0.05 and the number of case equals control’s, a total of 1042 MDD patients and 1042 healthy controls are intended to enroll. In view of 15 % data missing, a total of 1200 MDD patients and 1200 healthy controls should be included.

The second goal of this study is to identify potential indicators to assist MDD diagnosis. The study design for this part is a diagnosis test study. The sample size has been selected to detect small effects for indicators at area under receiver operating characteristic curve more than 0.6, with a power of 0.90 and a two-tailed significance level at 0.05 and the number of case is twice of control’s, a total of 260 MDD patients and 130 healthy controls are intended to enroll. In view of 15 % data missing, a total of 300 MDD patients and 150 healthy controls should be included. To confirm the performance of those indicators, another 300 MDD patients and 150 healthy controls should be included.

The third goal of this study is to identify factors which are associated with treatment outcomes of SSRIs on MDD. The study design for this part is a longitudinal-based case–control study. The sample size has been selected to detect small effects for factors at odds ratio 1.7, if the proportion of factors in control group more than 30 %, with a power of 0.82 and a two-tailed significance level at 0.05 and the number of case equal control’s (half of MDD patients who receive one of 6 SSRIs in this study would have treatment response at the end of week 8). A total of 520 MDD patients are intended to enroll. In view of 13 % data missing, a total of 600 MDD patients should be included.

### Data management

The data management center, basing on Java ezweb using B/S structure, has developed a web-based data system for data entering, checking and storage. After the paper-based questionnaire completed and checked, the site typist, with the typist’s ID and password, will input the data into the web-based data system. The system, using the regular express method to control the input format for different data type, will do the basic format and logic check when the typist inputting the data and will give tips to the typist if the data out of the logic range. After passing the system check, the typist will clicked “submit” and then all the entered original data will be transferred to database server and labeled questionable. The CRA, with the checker’s ID and password, will then review the questionable data, mark and write down their questions about the original data on the system and inform the researcher and the site typist to answer or modify the data, and then submit the reviewed data. After the reviewed data submitted, the data management center will lock and store the data in the final database using SQL Server 2008. The data system and database are installed in a host server which is collocated in an ISP server center and a special engineer is responsible for daily maintenance. Only having the approval of the executive management team, the engineer would export the database for the applicant. All data is de-identified using a unique ID number. The source documents are retained by each site and will be archived 10 years beyond study completion.

### Data preprocessing and statistical analysis

Systematically data cleaning and quantification will be done before analysis. In the process of data preprocessing, the most import step is to handle missing data. In this study, missing data will be filled with average value of same label samples. In view of distance calculation, techniques of data normalization and standardization will be used.

The statistical analyses would be performed using Stata 10.0 soft-ware. The details of analytic approach for each study aim are as follows:

1. Verify the role of heritability and environment in the onset of MDD and define the subtypes of MDD according to the influence of heritability and environment on its development.

According to classification hypothesis, significance test for the classified characteristics of 1200-MDD patients and1200-Healthy controls would be done. The confounding factors would be adjusted using regression models. The interaction between heritability and environment on the onset of MDD would be evaluated using logistic regression. Cluster analysis, K Nearest Neighbor and K-means algorithms would be used to analyze the demographic information, family history of psychiatric diseases, life events in the past year, adverse childhood experience, social support, coping style and personality trait and to explore if MDD would be classify several subtypes according to the influence of heritability and environment on its development. For the sake of further distinguish between this high dimensional data, fuzzy clustering and spectral clustering would be employed in this trail to distinguish subtypes of MDD.

2. Identify potential indicators to assist MDD diagnosis basing on the baseline information, neuropsychological assessment and biological markers.

The characteristics difference of 600-MDD patients subgroup and 300-Healthy controls subgroup will be analyzed. The correlation coefficient of each characteristic would be calculated and redundant features would be removed according to the experience of the clinical experts. Using logistic regression model to adjust the confounding factors, the effect of the rest factor set on outcome would be assessed and the main feature would be selected according to the weight coefficient. The principal component analysis and decision tree would be used to realize dimensionality reduction if the factor set is still large. To discriminate MDD patients from healthy controls, various classifiers, including support vector machines, decision tree, logistic regression and boosting, will be employed to cater to the demand of current situation. Significance test would be done for the selected factors and a characteristic will be considered an indicator if the p-value is <0.05. Half of 600-MDD patients subgroup and 300-Healthy controls subgroup would be used to train the model, and the rest half would be used to validate the model. The final indicators would be determined according to the best one model.

3. Identify overall predictors of treatment effectiveness on MDD after up to 8 weeks of SSRIs treatment and explore the predictors on MDD prognosis in the following two years.

Comparing with the baseline, according to the rate of decrease in HRSD17 at the end of 8-week, 600-MDD participants would be divided into effective group (the rate of decrease in HRSD17 ≥ 50 %) and ineffective group (the rate of decrease in HRSD17 < 50 %). The characteristics difference of the two groups will be analyzed. The correlation coefficient of each characteristic would be calculated and redundant features would be removed according to the experience of the clinical experts. Using logistic regression model to adjust the confounding factors, the effect of the rest factor set on outcome would be assessed and the main feature would be selected according to the weight coefficient. The principal component analysis would be used to realize dimensionality reduction if the factor set is still large. To predict treatment effectiveness, various classifiers, including typical classifiers such as support vector machines, decision tree, logistic regression and boosting and more powerful neural network, will be employed to cater to the demand of current situation. Significance test would be done for the selected factors and a characteristic will be considered a predictor if the p-value is <0.05. The final predictors would be determined according to the best one model. The analysis plan for exploring the predictors on MDD prognosis in the following two years is similar to aforementioned.

### Ethical issues

This study had been approved by the Ethic Committee of Peking University Sixth Hospital (approval NO. 2013-29-1). Written informed consent was obtained from each subject prior to undertaking any study-related procedures. They will be informed that they can withdraw from the study at any time without any negative consequence. To conform to data protection and freedom of information acts, all data will be stored securely and anonymised wherever possible. No published material will contain identifiable patient information. The study procedure is being monitored and is being re-assessed annually by the Ethic Committee of Peking University Sixth Hospital.

## Discussion

This study, a longitudinal, multi-stage and multi-center study, aims to verify the role of heritability and environment in the onset of MDD, define the subtypes of MDD, identify indicators to assist MDD diagnosis and assess predictors of treatment effectiveness of SSRIs on MDD. MDD may be divided into 3 types: heritability origin, stress origin and others. Potential indicators for diagnosis and predictors for treatment effectiveness may include demographic characteristics, stress events, clinical features, cognitive function, genotypes and peripheral biomarkers. Participants are recruited from clinical and academic sites to assemble a broadly inclusive and representative population. Thus, the study results should be widely generalization.

For different cause of MDD, the treatment response of antidepressants on MDD is usually various. To improve the treatment effectiveness, it is probably meaningful to divide MDD into different subtypes basing on its origin. To date, studies have typically examined one candidate marker at a time for assisting MDD diagnosis or predicting antidepressant response. By using standardized assessments to assess multiple candidate markers in the same study and same patients, this study provides enhanced statistical power to identify indicators to assist MDD diagnosis and assess predictors for antidepressant response. Promising preliminary empirical results coupled with recent developments in statistical methodology suggest that paradigms could be developed to provide useful clinical decision support in personalized treatment selection [47]. There will also be other strengths to this study, including strict quality control procedure, validated assessments by trained investigators, appropriate statistical analysis plan.

This study will have some limitations. First, brain imaging data was not collected owing to financial limit. Neuroimaging studies have demonstrated that brain imaging techniques could also assist to predict treatment outcomes of MDD [30, 48, 49]. On the other side, brain imaging techniques are quite complex and expensive at present and so it may be not suitable as a predictor for treatment response, especially in developing countries and regions. In this light, the predictors for treatment response on MDD studied in this study would be easily generalized. Second, the treatment for MDD patients is decided and adjusted by their attending physicians and they will be selected into this study if they meet the inclusion criteria. To some extent, the MDD subjects probably mirror clinical practice in a representative spectrum of MDD patients.

## Conclusion

MDD patients usually display a wide variation in clinical symptoms and sign, while the diagnosis of MDD is relatively subjective. A about half of MDD patients fail to respond adequately to the first antidepressant. The development of neurobiology and statistical methodology make it sense to combine a set of possible factors for assisting the diagnosis and improving treatment effectiveness of MDD. The results of this study should provide strong and suitable evidence for enhancing the accuracy of MDD diagnosis and promoting personalized treatment for MDD in clinical practice.

## Trial status

The recruitment of participants started in December 2013 and will stop at December 2016.

### Role of the funding source

This project is funded by the National Key Basic Research Program of China (No.2013CB531305). The study protocol has undergone peer-review by the funding body.
